# Endoscopic submucosal dissection by using a new traction device

**DOI:** 10.1016/j.vgie.2021.08.010

**Published:** 2021-10-27

**Authors:** Masami Omae, Naining Wang, J-Matthias Löhr, Miroslav Vujasinovic, Francisco Baldaque-Silva

**Affiliations:** 1 Division of Medicine, Department of Upper Gastrointestinal Diseases, Karolinska University Hospital and Karolinska Institute, Stockholm, Sweden; 2 Department of Pathology, Karolinska University Hospital and Karolinska Institute, Stockholm, Sweden; 3 Division of Medicine, Department of Upper Gastrointestinal Diseases, Karolinska University Hospital and Karolinska Institute, Stockholm, Sweden

**Keywords:** ESD, endoscopic submucosal dissection

## Abstract

Video 1 Patient with a history of gastric ectopic pancreas and epigastric pain. We illustrate the endoscopic submucosal dissection of the ectopic pancreas using a new traction device, the ProdiGi traction wire. Using this device, we were able to resect the lesion en bloc with no adverse events.

Patient with a history of gastric ectopic pancreas and epigastric pain. We illustrate the endoscopic submucosal dissection of the ectopic pancreas using a new traction device, the ProdiGi traction wire. Using this device, we were able to resect the lesion en bloc with no adverse events.

Several devices and techniques have been described to assist endoscopic submucosal dissection (ESD), but all have their limitations. Most enable only traction,^1^ some are invasive^2^ or complex,^3^ and others demand several steps.^4^ We describe the use of a new traction wire device (ProdiGi Traction Wire, Medtronic, Minneapolis, Minn, USA) that is easy to deploy and enables easier access to the submucosa during ESD.

A 26-year-old man with a long history of severe epigastric pain was referred to our clinic. His blood tests were unremarkable. A gastroduodenoscopy showed the presence of a 20-mm submucosal tumor in the greater curvature of the antrum, with normal mucosa and central umbilication, suggestive of ectopic pancreas (Fig. 1). An EUS revealed a heterogeneous lesion, with a “salt and pepper” pattern, engaging the submucosa with poor demarcation from the muscularis propria, with cystic areas, a duct, hyperechoic foci, and stranding (Fig. 2). These findings were compatible with ectopic pancreas showing signs of chronic pancreatitis.

**Figure 1:**
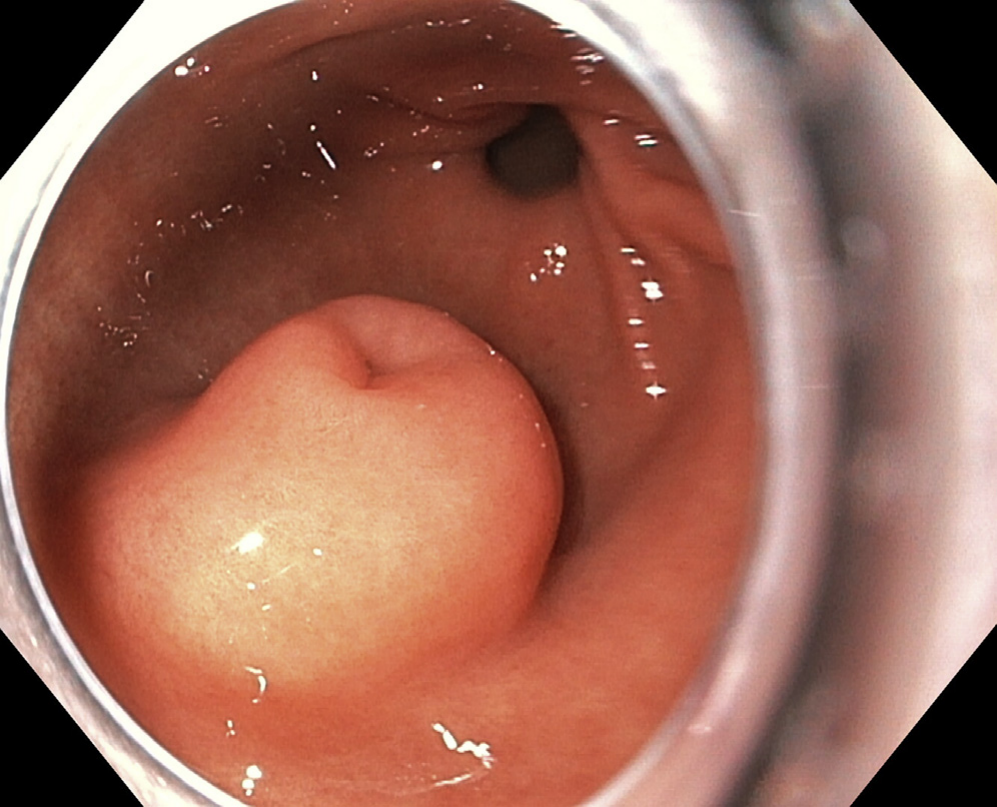
An EGD shows a submucosal tumor with 20-mm diameter in the greater curvature of the antrum, with normal mucosa and central umbilication, suggestive of ectopic pancreas.

**Figure 2:**
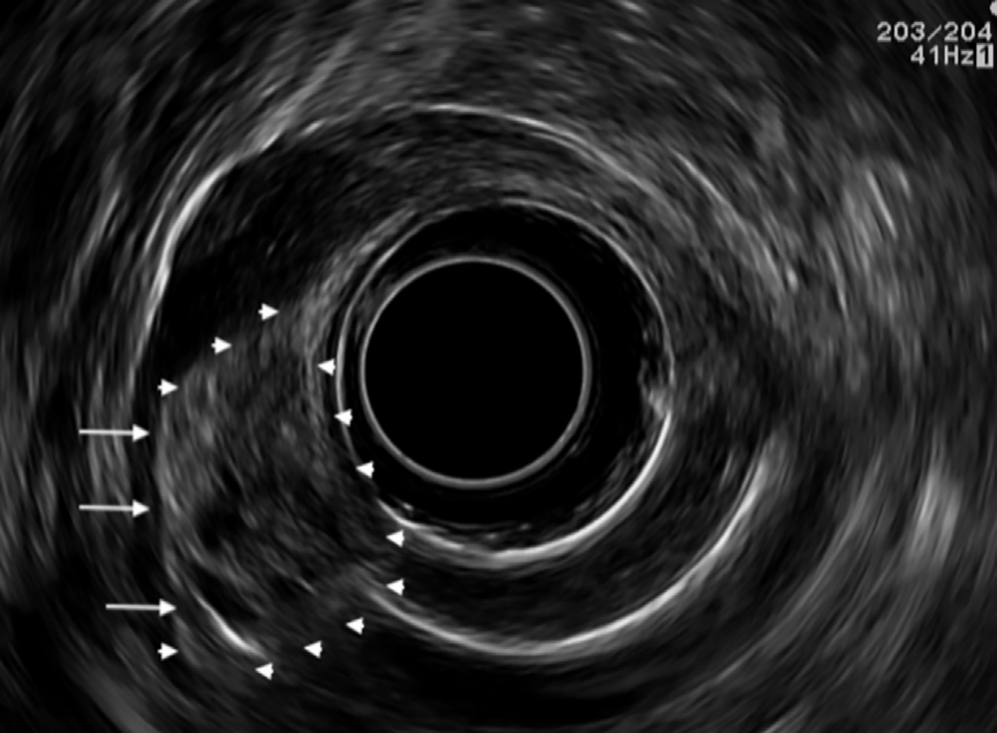
EUS reveals a heterogeneous lesion, with salt and pepper pattern, engaging the submucosa (*arrowheads*), with poor demarcation from the muscularis propria (*arrows*) and with cystic areas, a duct, and hyperechoic foci and stranding. These findings are compatible with ectopic pancreas with signs of chronic pancreatitis.

Because of the patient’s recurrent episodes of epigastric pain, the case was discussed in a multidisciplinary conference. A decision for ESD resection was made, and the procedure was performed after the patient provided informed consent.

A gastroscope (GIF-HQ190J; Olympus, Hamburg, Germany), a 2.0-mm ESD knife (ProdiGI knife, Medtronic), and a lifting gel (Orise gel, Boston Scientific, Marlborough, Mass, USA) were used. Butylscopolamine was administered during the procedure to reduce GI motility.

Marking dots were placed with the tip of the knife (Fig. 3). Initial subepithelial injection and incision were performed distally, followed by proximal injection and incision (Fig. 4). The dissection from the muscularis propria was difficult, as expected, and we used a 35-mm traction wire to perform it. This system has 2 components: a primary clip with a traction nitinol wire and a secondary clip to secure the distal end of the wire (Fig. 5). First, the primary clip and wire were inserted through the 2.8-mm working channel and the traction wire was deployed onto the targeted tissue (Fig. 6). Next, the secondary clip was inserted and used to grasp and secure the free end of the wire to the targeted wall (Fig. 7). It was difficult to dissect the lesion from the muscularis propria; however, this device provided continuous tension during the ESD, enabling full resection with free margins without bleeding or muscular damage. The specimen was retrieved with the traction wire and clips, with no trauma in the esophagus or oropharynx. The pathology analysis showed the presence of a fully resected ectopic pancreas with extensive areas of chronic inflammation, fibrosis, and mild atypia (PanIN 1A, Fig. 8). The patient was discharged with no postoperative adverse events (Video 1, available online at www.VideoGIE.org).

**Figure 3:**
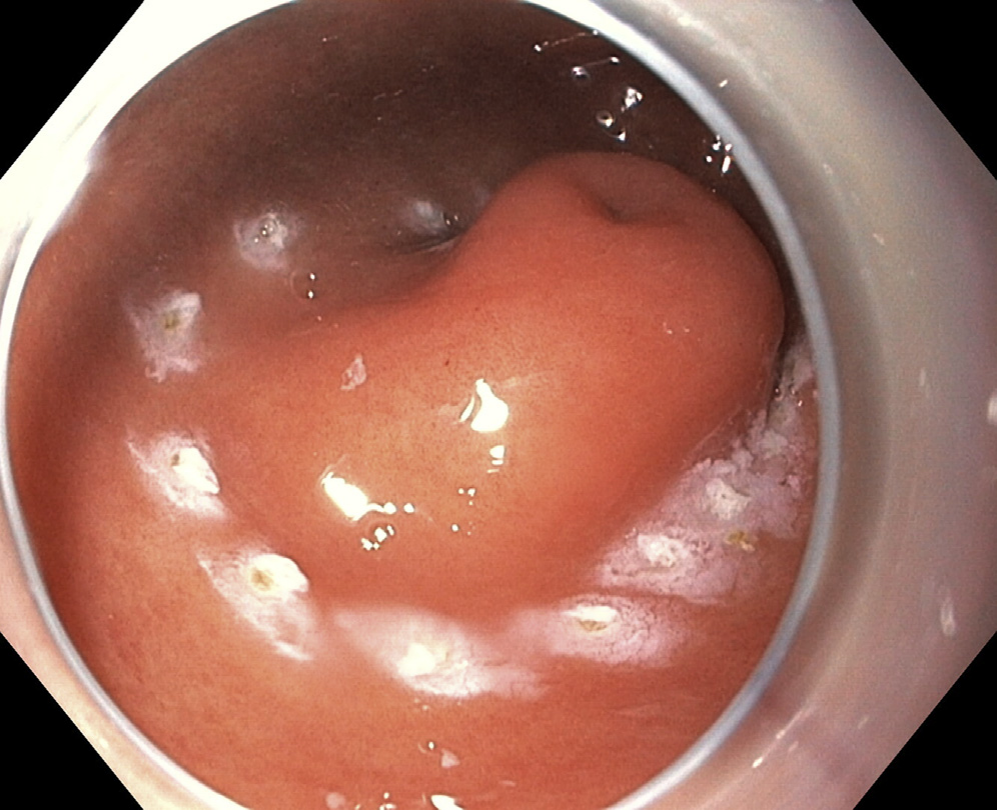
Marking dots are placed with the tip of the knife.

**Figure 4:**
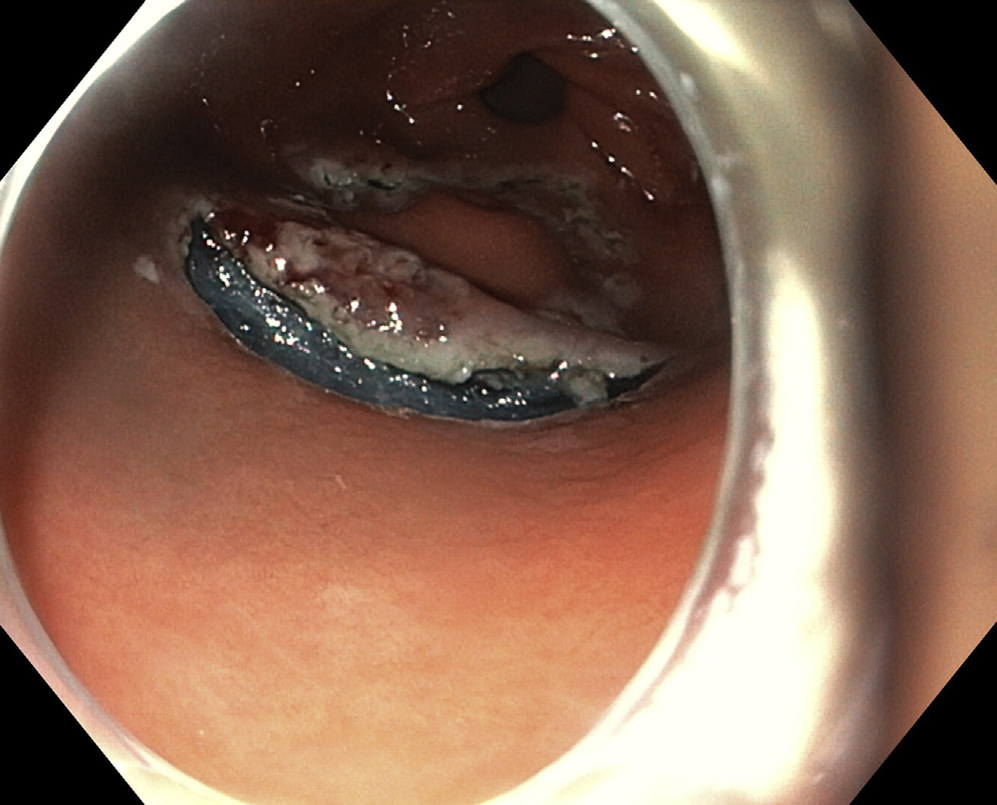
Initial subepithelial injection and incision are performed distally, followed by proximal injection and incision.

**Figure 5:**
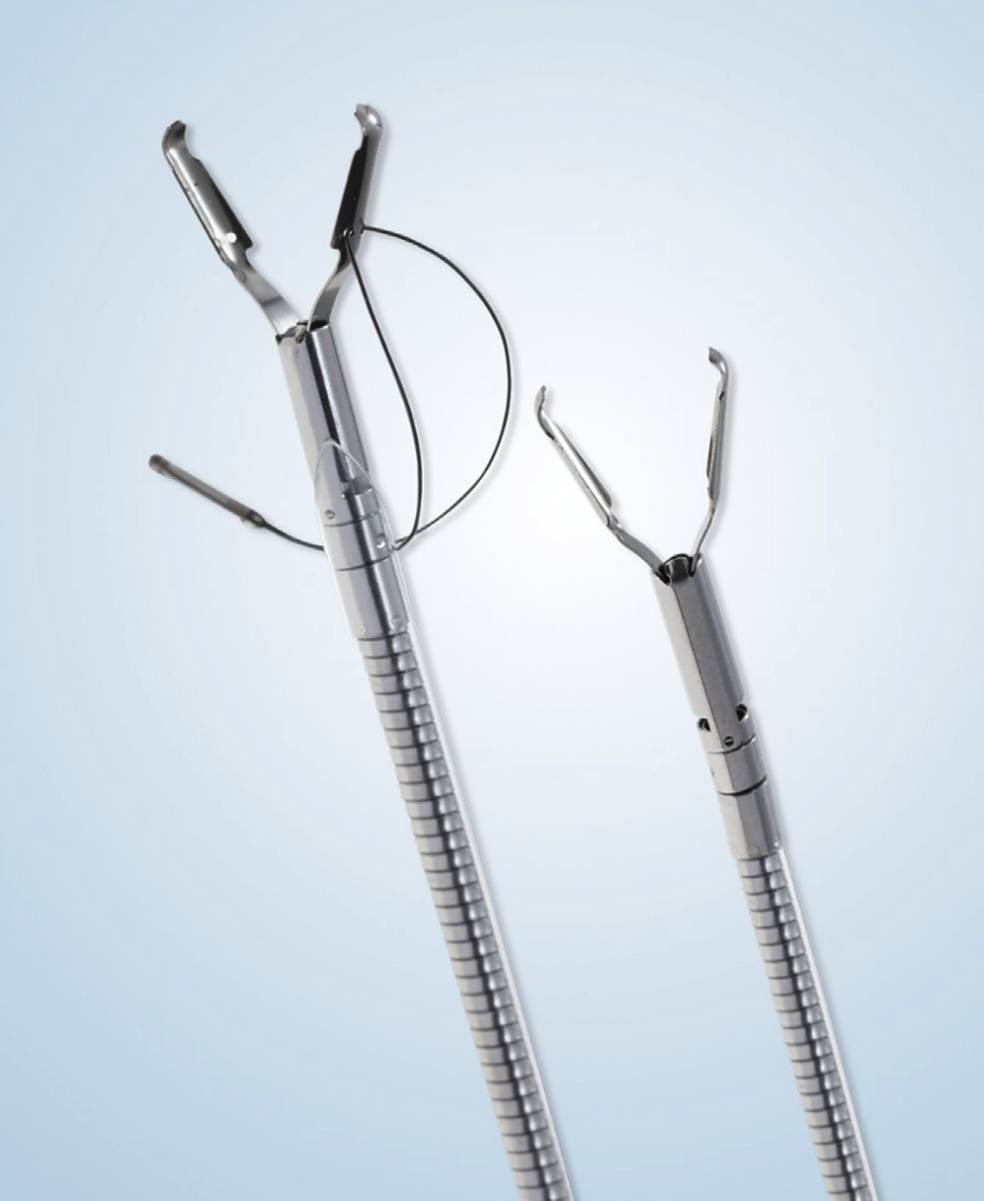
This is a ProdiGi Traction Wire (35 mm) device. This system has 2 components: a primary clip with a traction nitinol wire and a secondary clip to secure the distal end of the wire.

**Figure 6:**
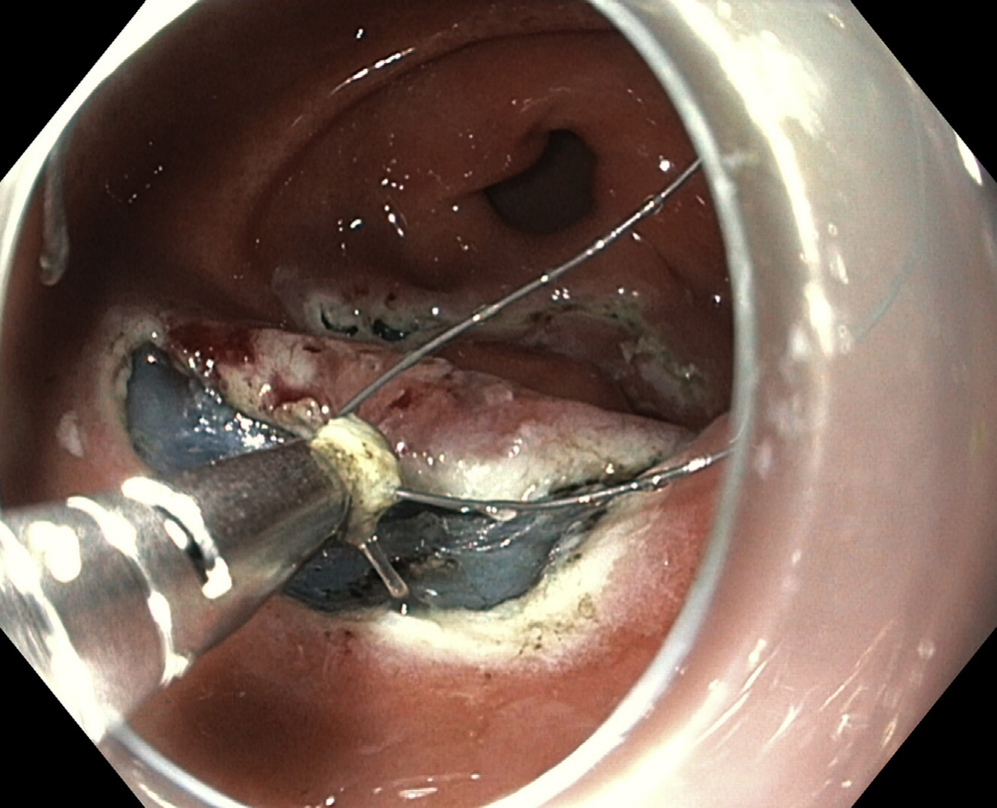
The primary clip with wire is deployed onto the targeted tissue.

**Figure 7:**
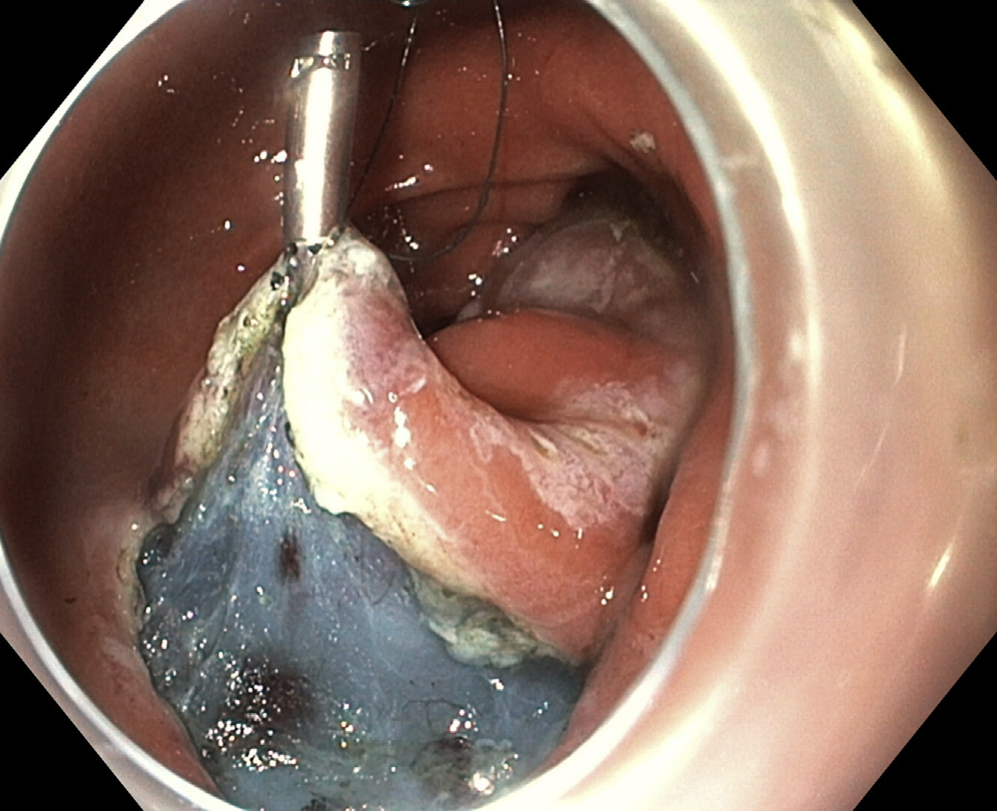
The second clip is used to grasp and secure the free end of the wire to the targeted wall.

**Figure 8:**
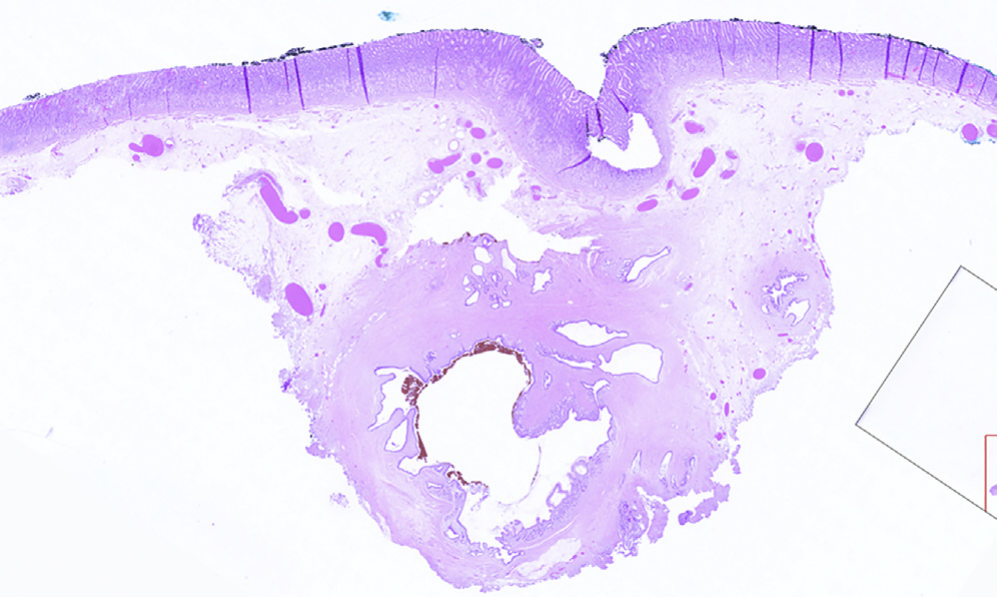
The pathologic investigation demonstrates the presence of a fully resected ectopic pancreas with extensive areas of chronic inflammation, fibrosis, and mild atypia (PanIN 1A).

Gastric ectopic pancreas is typically located in the greater curvature of the distal antrum and usually grows from the submucosa, but it might engage the mucosa or proper muscle layers.^5^ Most patients are asymptomatic and require no treatment or follow-up. Resection is recommended in rare cases of pancreatitis,^6^ bleeding,^7^ and malignant transformation.^8^ Our patient had episodes of severe epigastric pain and EUS findings suggestive of chronic pancreatitis. ESD was performed after a multidisciplinary conference and discussion with the patient. The epigastric pain ceased with the resection, and the patient remains asymptomatic at 3 months’ follow-up.

We report a technically difficult gastric ESD owing to strong adherence between the ectopic pancreas and muscularis propria. ESD was safe and successful using a new traction wire. This device has specific characteristics: (1) because of the thermal memory of the nitinol wire, this device maintains its half-moon shape, enabling continuous tension through the procedure; (2) the site of mucosal traction can be modified by removing the secondary clip and placing a new one in another location in the wall; (3) it is a dynamic technique in which additional secondary clips can be used if desired to adjust the position during the ESD; (4) internal traction can be obtained (ie, there is no need for the use of strings or snares through the nose or mouth); and (5) there is no need for extra personnel to handle the device after its deployment.

In conclusion, this traction system is easy to deploy, user-friendly, and useful for difficult ESDs. Further comparative studies with other techniques or devices are warranted.

## Disclosure

*All authors disclosed no financial relationships*.

## Supplementary data

Video 1 Patient with a history of gastric ectopic pancreas and epigastric pain. We illustrate the endoscopic submucosal dissection of the ectopic pancreas using a new traction device, the ProdiGi traction wire. Using this device, we were able to resect the lesion en bloc with no adverse events.
